# Anaphylaxis to IGIV in immunoglobulin-naïve common variable immunodeficiency patient in the absence of IgG anti-IgA antibodies: successful administration of low IgA-containing immunoglobulin

**DOI:** 10.1186/s13223-016-0132-2

**Published:** 2016-05-17

**Authors:** Asal Gharib, Caroline Caperton, Sudhir Gupta

**Affiliations:** Division of Basic and Clinical Immunology, Medical Sciences I, C-240, University of California at Irvine, Irvine, CA 92697 USA

**Keywords:** CVID, Anaphylaxis, IVIG, Case report

## Abstract

Although severe reactions to immunoglobulin preparations have been frequently reported, IgE antibodies against IgA are usually not investigated; and occur predominantly in previously sensitized patients. The purpose is to report anaphylaxis to IGIV during initial infusion in a patient with common variable immunodeficiency with absent IgA without prior sensitization and in the absence of detectable IgG anti-IgA antibodies, and positive skin tests for immediate hypersensitivity to four different preparations of IGIV, one subcutaneous immunoglobulin preparation, and to purified IgA. Patient was treated without side effects with IGIV preparation depleted of IgA to which immediate hypersensitivity skin test was negative.

This case demonstrates that patients with CVID with no IgA and without prior exposure to immunoglobulin or plasma may develop anaphylaxis following initial infusion of IGIV, which appears to be due to IgE anti-IgA, and independent of IgG anti-IgA antibodies. Since there is no good correlation between anaphylaxis/anaphylactic reactions and IgG anti-IgA antibodies, and IgE anti-IgA antibody test is commercially unavailable, we suggest that the patients with CVID with absence of IgA might be skin tested for immediate hypersensitivity prior to initiation of immunoglobulin administration. However, such recommendation may require studies on a large number of patients with CVID with no detectable IgA.

## Background

Common variable immunodeficiency (CVID) is a heterogeneous disorder characterized by decreased levels of at least two immunoglobulin isotypes, including IgG and impaired specific antibody response [1]. Anaphylactic/anaphylactic reactions to blood and plasma transfusion have been reported in subjects with IgG anti-IgA antibodies [2, 3]. Immunoglobulin is the standard of care for antibody deficiency syndromes. Current practice does not specify the use of any specific immunoglobulin preparation or testing prior to infusion. Infrequently patients develop systemic sensitivity to intravenous immunoglobulin (IGIV) treatment, including anaphylaxis/anaphylactic reactions, which may be associated with IgG-anti-IgA antibodies and often associated with prior exposure to immunoglobulin therapy [4, 5]. In contrast, patients with hypogammaglobulinemia and IgG anti-IgA antibodies have tolerated immunoglobulin therapy without any reaction [6]. Therefore, there is a lack of correlation between IgG anti-IgA antibodies and anaphylactic/anaphylaxis reaction [4, 7]. A few cases have been reported in whom IgE-mediated anaphylaxis developed following immunoglobulin therapy [4, 8]. However, these patients were receiving and tolerating immunoglobulin prior to development of anaphylaxis. We report a patient with common variable immunodeficiency (CVID) with no detectable IgA and naïve to immunoglobulin therapy, who developed what appears to be IgE anti-IgA antibody mediated, and IgG anti-IgA independent anaphylaxis during initial 5 min of first IGIV infusion.

## Case description

A 37 year-old male with a history of chronic ear infections throughout childhood, and childhood asthma and rhinitis was referred to immunology clinic for an evaluation. Since the age of 15 years, he is having 2–3 episodes of bronchitis per year, developed recurrent otitis media requiring myringotomy tubes, and suffered six image-proven sinus infections in the previous 2 years. In 2013, he was hospitalized twice for pneumonia. He has no history of receiving prior blood or blood product transfusions. In 8/2014, a diagnosis of common variable immunodeficiency (CVID) was confirmed. His laboratory findings are shown in Table 1. Gammagard 10 % IV infusion was started at the rate of 30 cc/h. Within 5 min of starting IGIV, patient reported tightness of chest, difficulty in breathing, facial flushing, bilateral wheezes, pulse rate of 126/min, severe rigor, and oxygen saturation drop to 86 %. Patient was given IV famotidine, IV meperidine, IV hydrocortisone, IV Benadryl, and Oxygen at 6 L. Patient was skin tested for various IGIV and subcutaneous Ig preparations along with purified IgA and normal saline. On 2/11/2015, Patient was given graded dosage of IgA-depleted IGIV (Gammagard SD) preparation (5, 10, 30 ml) to which skin test was negative, with premedication of prednisone, Benadryl, and acetaminophen orally an hour prior to infusion. He tolerated the infusion without any side effects. Patient tolerated infusion at 80 ml/h rate. Patient has no history of allergies to pollen, food, or drugs.

**Table 1:** Immunological profile of the patient

Laboratory test	Patient’s value	Reference value
IgG	<33 mg/dl	694–1618 m/dl
IgA (mg/dl)	<6.7	68–378
IgM (mg/dl)	<4.2	65–263
IgE (IU/ml)	<1	10–150
IgG anti-IgA antibody (U/ml)	<16	<99
Complement Total, CH50 (U/ml)	137	101–300
Complement, C3 (mg/dl)	120	88–201
Complement, C4 (mg/dl)	27	16–47
Total WBC count	8000/^3^ml	4000–10,500/^3^ml
Lymphocytes, %	25	14–44
Total lymphocytes	2000/^3^ml	900–3300/^3^ml
CD3 + CD4+ %	37	31–61
CD3 + CD4+	740/^3^ml	338–1194/^3^ml
CD3 + CD8+ %	35	10–38
CD3 + CD8+	700/^3^ml	85–729/^3^ml
CD4: CD8 ratio	1.06	0.9–3.7
CD3, %	78	62–84
CD3, total	1560/^3^ml	619–1847/^3^ml
CD19, %	17	5–26
CD19, total	340/^3^ml	51–473/^3^ml
CD56, %	4	1–17
CD56, total	80/^3^ml	12–349/^3^ml

## Methods and results

This study was approved by the Institution Review Board (Human), University of California, Irvine. Consent was obtained from the patient.

Serum immunoglobulins, complement levels, and IgG anti-IgA antibodies were performed were Department of Pathology, University of California, Irvine. Serum Immunoglobulins were performed by nephelometry. Lymphocyte subsets were performed with anti-CD3, anti-CD4, anti-CD8, anti-CD19, anti-CD16, and anti-CD56 monoclonal antibodies and corresponding isotype controls (Pharmingen BD Sciences, San Jose, California) using FACSCalibur (Becton–Dickinson, San Jose, California). 10,000 cells were acquired and analysis was performed with FlowJo software (Treestar, Ashland, Oregon). Patient has extremely low levels of all immunoglobulin and normal functional CH50 and normal levels of CD3 and C4 complements. T cells, T cell subsets, B cells and natural killers are present in normal proportions and numbers.

Because patient has a true anaphylaxis and had undetectable serum IgG anti-IgA antibodies, it was reasoned that his anaphylaxis was most likley IgE-mediated. Patient was skin tested for immediate hypersensitivity with four different IGIV preparations and one immunoglobulin subcutaneous preparation (IGSC). All preparations except Octagam (1:10 dilution because high IgA content) were used undiluted. Furthermore to establish that immediate hypersensitivity reactions are against IgA in various preparations, skin tests were also performed with purified IgA (Sigma, St. Louis, MO) and preparation of IGIV that has negligible amount of IgA.

Data in Table 2 demonstrates positive wheal/flare reactions to various IGIV and IGSC preparations, indicating IgE-mediated sensitivity to Immunoglobulin preparations. Furthermore, patient had positive wheal/flare reaction within 15 min to purified human IgA indicating that positive reactions to all commercially available preparations tested were most likely due to IgE anti-IgA antibodies. The patient was then skin tested with Gammagard SD (very low IgA) and no local reaction was observed, similar to normal saline.

**Table 2:** Patient’s immediate hypersensitivity intradermal skin tests

Immunoglobulin preparations	Wheal (in mm)	Flare (in mm)
Gammagard liquid 37 ug/ml	9	58
Hizentra 50 ug/ml	18	80
Privigen 25 ug/ml	21	78
Octagam 200 ug/ml	10	60
Gammagard-S/D <1 ug/ml	2	3
Purified human IgA 10 ug/ml	10	25
Normal saline	3	4

## Discussion

Anaphylaxis upon first exposure is rare, and generally requires sensitization via prior exposure to an antigen, or a structurally related antigen, which may induce cross-reactivity. Patients with CVID who have a history of severe adverse reactions to IGIV therapy most frequently are those with undetectable levels of IgA. The pathophysiology is attributed to immune complexes of IgG anti-IgA antibodies resulting in complement activation and manifesting as anaphylaxis/anaphylactic reactions upon exposure to IGIV therapy, which includes variable concentrations of contaminating IgA [9].

The prevalence of IgG anti-IgA antibodies in patients with CVID has been reported as high as 9 % [10]. A patient with CVID, who tolerated immunoglobulin intramuscular for 6 years and two IGIV infusions, developed a severe anaphylactic reaction during third IGIV infusion [11]. This was associated with the presence of IgG anti-IgA antibodies, immune complexes, and evidence of complement activation. This patient tolerated IgA-depleted IGIV. However, there is no good correlation between IgG anti-IgA antibodies and anaphylactic reaction; IgG anti-IgA antibodies have been observed in CVID patients with undetectable IgA [12] and normal IgA [7] without any systemic reaction to IGIV. In our patient, IgG anti-IgA antibodies were absent, and CH50, C3, and C4 were normal; however, we did not perform C3 split products to completely exclude a possibility of complement activation. Since our patient has no IgG anti-IgA antibodies, therefore, unlikely to have circulating immune complexes to activate complement. Furthermore, IgE does not fix the complement, therefore, immune complex-mediated activation of complement appears to be unlikely a mechanism for anaphylaxis in our patient. Finally, complement-mediated activation of mast cell is associated with delayed skin induration and flare and not immediate as observed with IgE-mediated immediate hypersensitivity.

True IgE-mediated anaphylaxis to IGIV preparations is exceedingly rare and usually requires prior sensitization. Since IgE antibodies against IgA are usually not investigated, frequency of IgE-mediated anaphylaxis to IVIG in CVID is not known.

Burks and colleagues [8] reported anaphylaxis in two patients with CVID with absence of IgA. First patient developed anaphylaxis after 5th intramuscular injection of immunoglobulin (prior sensitization), and second patient developed anaphylaxis following initial infusion of plasma from the patient’s father, which would contain normal levels of IgA, almost 100 fold higher than immunoglobulin preparations. Both serum IgG anti-IgA and IgE anti-IgA antibodies were detected by ELISA assay. However, presence of IgE anti-IgA antibodies in one of these two patients could not be replicated [12]. Ferreira et al. [13] reported presence of a IgE anti-IgA in a patient with CVID, who later developed anaphylaxis with IGIV. In another study, Ferreira and associates [7] reported presence of IgE anti-IgA antibodies in a patient with hyper IgM syndrome; however, it was unclear whether this patient received immunoglobulin therapy and if received how he/she tolerated it.

Our patient was distinct from all these patients in that he was naïve to immunoglobulin, and had no circulating IgG anti-IgA antibodies. Finally, he had positive immediate hypersensitivity skin test reactions to four different preparations of IGIV, one subcutaneous Immunoglobulin, and to purified IgA, establishing that his anaphylaxis was most likely IgE anti-IgA-mediated. However, surprisingly we did not find correlation between the size of wheel or flare with the concentrations of IgA in various IGIV and IGSC preparations. Immediate hypersensitivity skin test reaction was negative to Gammagard S/D (very low concentration of IgA), a preparation, which he later tolerated given intravenously, and normal saline. Interestingly, our patient has very low serum IgE, which suggest that either traces of serum IgE has anti-IgA activity or most of IgE anti-IgA antibodies are mast cell associated, and very little free antibody in the serum. It has been demonstrated that a single glycan on IgE is indispensible for initiation of anaphylaxis [14]. This patient highlights that anaphylaxis can occur in the presence of very low serum IgE. A possibility of the presence of possible mast cell-associated IgE anti-IgA in the absence of circulating IgE anti-IgA might also explain a lack of correlation between size of wheal and flare reactions with preparations of immunoglobulin with various concentrations of IgA.

Limaye et al. [15] reported a patient with CVID and undetectable levels of IgA, who developed anaphylaxis twice to blood products. Prior to starting IGIV, patient was skin tested with two different IVIG concentrations and report positive skin test with IGIV products containing high concentrations of IgA but negative skin to a product with very low concentrations of IgA. They also did not measure IgE anti-IgA antibodies. Patient with Burk et al. [8] also had both IgG anti-IgA and IgE anti-IgA antibodies. In our patient, there were no IgG anti-IgA antibodies.

Bjorkander et al. [12] observed IgG anti-IgA antibodies in 74 immunodeficiency patients with undetectable IgA, 8 of them received low IgA containing IGIV (IgA <20 ug/ml), one of the patients developed anaphylaxis, whereas other tolerated IGIV infusions. None of them had IgE anti-IgA. It is possible that skin test for immediate hypersensitivity may provide more sensitive test than ELISA or radioimmunoassay; this would be especially true for patient, who may have predominantly mast cell-associated IgE anti-IgA antibodies with negligible amounts in the serum. It is well known that skin tests are more sensitive than serological immunoassays for allergens. The precise sensitivity of these immunoassays compared with prick/puncture skin tests has been reported to be approximately 70–75 %. In most situations, skin tests are therefore the most clinically useful tests for the diagnosis of IgE-mediated sensitivity [16]. It is unlikely that positive skin tests were due to an non-specific irritation. Control saline and Gammagard S/D were negative in the patient. We have performed skin tests in three other patients with CVID with negative results.

Since there is no good correlation between IgG anti-IgA antibodies and anaphylaxis, no recommendation to screen for these antibodies, and no commercially available IgE ELISA assay for IgE anti-IgA antibodies, we propose that patients with CVID and absent IgA may be skin tested for immediate hypersensitivity with immunoglobulin preparation prior to initial infusion. In those patients with positive reactions, immunoglobulin preparation with very low IgA content may be used. It can be argued that why not administer Gammagard-SD in immunodeficiency patients with undetectable IgA without going through skin testing. The reason is that Gammagard-SD comes in the powder form and has to be reconstituted under strict sterile condition, which may not be available in outpatient or private office settings. Furthermore, Gammagard-SD contains sugar as preservative, has high osmolarity, and hence, greater possibility of renal side effects of IGIV. Therefore, use of Gammagard-SD should be restricted to those patients, who demonstrate positive skin test to immunoglobulin preparation to be used.

## Conclusions

We suggest that the patients with CVID with absence of IgA, even those naïve to immunoglobulin or plasma products, may be skin tested for immediate hypersensitivity to immunoglobulin preparations prior to initiation of immunoglobulin administration. In case of positive skin test, an IgA-depleted preparation of immunoglobulin may be used to minimize a possibility of anaphylactic reaction. However, a study of large number of patients with CVID and undetectable IgA is needed prior to making it as a universal recommendation.
